# A multicenter clinical evaluation of *Mycobacterium tuberculosis* IgG/IgM antibody detection using the colloidal gold method

**DOI:** 10.1007/s10096-014-2150-7

**Published:** 2014-06-10

**Authors:** Y. Wang, B. Lu, J. Liu, T. Xiao, K. Wan, C. Guan

**Affiliations:** 1 Department of Physiology, Xiangya School of Medicine, Central South University, Changsha, Hunan 410078 China; 2 National Institute for Communicable Disease Control and Prevention, Chinese Center for Disease Control and Prevention, State Key Laboratory for Infectious Disease Prevention and Control, P.O. Box 5, Changping, Beijing, 102206 China; 3 Collaborative Innovation Center for Diagnosis and Treatment of Infectious Diseases, Hangzhou, 310003 China; 4 China-Japan Friendship Hospital, Yinghua East Street, Beijing, 100029 China

## Abstract

A specific immunoassay method with the colloidal gold labeling technique has been developed more and more for tuberculosis (TB) diagnosis. The aim of this multicenter clinical evaluation was to evaluate the performance of a new serological diagnostic kit (the Trustline TB IgG/IgM Rapid Test kit) for the detection of *Mycobacterium tuberculosis* infection in China, with the Aupu TB Ab (IgG) Colloidal Gold Test kit being used as a control. A total of 1,009 specimens were collected from three TB hospitals, including 628 patients with TB, and 219 non-TB and 162 healthy patients as negative controls. According to the clinical diagnostic results, the sensitivities of the Trustline kit and the Aupu kit were 61.3 % and 53.7 %, respectively. Using the bacteriological test results as the “gold standard” to compare the results of the two kits, the sensitivity, specificity, positive predictive value (PPV), negative predictive value (NPV), and Youden index (YI) were 77.60 %, 79.8 %, 73.31 %, 81.50 %, and 0.574 by the Trustline kit, and 67.86 %, 88.9 %, 83.27 %, 77.40 %, and 0.568 by the Aupu kit, respectively. Further, the sensitivity of the Trustline kit and the Aupu kit for the smear staining and the bacterial culture being positive was 75.6 %, 76.6 % and 65.6 %, 66.5 %, and for the negative result, it was 53.8 %, 50.9 % and 47.5 %, 45.0 %, respectively. Additionally, 35 specimens were IgM-positive by the Trustline kit; of these, 30 (4.8 %) were from patients with TB and 5 (1.3 %) were from individuals without TB. The results showed that the experimental test had a much higher sensitivity than the other commercial test and exhibited a good detection rate for *M. tuberculosis* infection. Therefore, this kit can be used in the supplementary diagnosis and screening of TB.

## Introduction

It is reported that tuberculosis (TB) caused by *Mycobacterium tuberculosis* (*M. tuberculosis*) infection has become one of the major fatal infectious diseases among adults in the world [1]. China is one of the 22 countries in the world having a high TB burden and has the second highest number of active TB cases, with more than 1 million new cases of TB diagnosed each year.

Currently, patients are diagnosed with TB based mainly on clinical symptoms, X-rays, and conventional laboratory tests, such as bacterial cultures and sputum smear acid-fast staining, which exhibit much lower sensitivity. Acid-fast staining cannot be used for the diagnosis of extrapulmonary TB, such as tuberculous pleurisy, tuberculosis of lymph nodes, tuberculosis meningitis, etc., and bacterial culture is a time-consuming method that is unsuitable for rapid and early diagnosis, and it also has very low sensitivity. Therefore, researchers are interested in the development of new rapid-detection approaches, such as immunological [2] and nucleic acid [3] detection techniques.

Since the 1980s, a number of new immunoassay techniques has been developed using three labeling techniques (fluorescein, radioisotopes, and enzymes) [4]. Such methods were initially used only for immunoelectron microscopy; however, over time, these methods have been used for additional applications in passive agglutination tests, light microscopy staining, immunoblotting, immunoblot filtration assays, and immunoassays [5–7]. These techniques use chromatography membranes precoated with specific substrate(s) as the solid phase; as the sample solution moves through the membrane by means of capillary action, the analytes in the sample react with specific substrates with high affinity. The resulting immune complexes are enriched or trapped within the membrane and can be visualized with markers, such as colloidal gold. This technology does not require special equipment, the products have a long shelf life and can be stored at room temperature, the operators do not need special training, and results can be obtained within 10–20 min and observed by the naked eye, making the immune colloidal gold technique especially suitable for the majority of rural and community clinics and local hospitals, on-scene investigators, large-scale urgent testing, and general disease surveys [4, 8–12].

In this clinical trial, we evaluated the performance of the Trustline kit (produced by Beijing Genesee Biotech, Inc.) against the Aupu kit, a dot immunogold filtration assay (DIGFA) [13]. The Trustline kit, which can detect *M. tuberculosis* IgG/IgM antibodies, uses a colloidal gold immunochromatography method. It uses four recombinant *M. tuberculosis* protein antigens (6, 14, 16, and 38 kDa) simultaneously in a single-step procedure that is simple to carry out and provides rapid results.

## Materials and methods

This research was approved by the Ethics Committee of the National Institute for Communicable Disease Control and Prevention, Chinese Center for Disease Control and Prevention, Beijing, China. All the patients included in this study provided signed informed consent to participate in the investigation.

In this study, the experimental kit was the Trustline TB IgG/IgM Rapid Test kit (Beijing Genesee Biotech, Inc., Beijing, China). The control kit was an *M. tuberculosis* IgG antibody colloidal gold kit, the Aupu TB Ab (IgG) Colloidal Gold Test kit (Shanghai Aupu Biotechnology Co., Shanghai, China), which had been approved by the China Food and Drug Administration (CFDA, authorization code 20030090).

A total of 1,009 participants’ serum samples were collected from three hospitals (Table 1), among which 628 TB samples formed the case group, including 539 pulmonary TB and 89 extrapulmonary TB patients, and 381 samples comprised the negative controls, including 162 medical examiners (healthy) and 219 patients with non-TB lung diseases (non-TB). The patients had an average age of 43 ± 1.96 years, and the gender ratio (male/female) was 1.69/1. The demographic information for the 1,009 participants is shown in Table 2. All of the 1,009 participants were negative for human immunodeficiency virus (HIV)/acquired immunodeficiency syndrome (AIDS) diagnosed with the China national diagnostic criteria and principles of management of HIV/AIDS. The antibodies against HIV in human plasma was tested with a third-generation HIV antibody enzyme-linked immunosorbent assay (ELISA) test, Vironostika HIV-1/2 Microelisa System (bioMérieux, Holland), within 24 h, according to the manufacturer’s instructions [14].

**Table 1:** Distribution of serum samples in the three different hospitals in China

Samples	Samples, *n* (%)			Total, *n*
	TB	Non-TB	Healthy	
Beijing Geriatric Hospital (Beijing)	241 (72.8)	36 (10.9)	54 (16.3)	331
General Hospital of Huabei Oil Field Company	188 (53.7)	105 (30.0)	57 (16.3)	350
Cangzhou Infectious Disease Hospital (Hebei Province)	199 (60.7)	78 (23.8)	51 (15.5)	328
Total	628 (62.2)	219 (21.7)	162 (16.1)	1,009

**Table 2:** Demographic information for the 1,009 participants

		Pulmonary TB (%)	Extrapulmonary TB (%)	Non-TB (%)	Healthy	Total
Total		539 (63.4)	89 (8.8)	219 (21.7)	162 (16.1)	1,009
Gender	Male	351 (60.2)	56 (9.6)	140 (24.0)	86 (14.8)	583
	Female	188 (50.0)	33 (8.8)	79 (21.0)	76 (20.2)	376
Age	Mean ± SD	45 ± 1.83	38 ± 1.78	39 ± 2.08	35 ± 1.67	43 ± 1.96
	≤14	7 (25.0)	3 (10.7)	6 (21.4)	12 (42.9)	28
	>14	532 (54.2)	86 (8.8)	213 (21.7)	150 (15.3)	981

Diagnoses of pulmonary TB in the study were made with the Clinical Diagnosis Standard of TB for Clinical Technology Operation (TB volumes) of the Chinese Medical Association published by the People’s Medical Publishing House (PMPH; ISBN 9787117065108). Diagnoses of extrapulmonary TB were made with the Guideline of the Ministry of Health of China. In this study, we judged the final diagnosis of all patients with curative effects.

We used the two kits (Trustline and Aupu) to detect antibodies against *M. tuberculosis* in the sera samples in accordance with the respective manufacturer’s instructions. We compared the kits using the sensitivity, specificity, positive predictive value (PPV), negative predictive value (NPV), Youden index (YI), and negative and positive bacteriology rates.

The bacteria from the pulmonary TB patients were detected by means of sputum smear acid-fast staining and *Mycobacterium* culture on Löwenstein–Jensen medium [15].

The data were analyzed using SPSS statistical software (version 16.0, SPSS Inc., Chicago, IL, USA). We used Cohen’s kappa (κ) equivalence test to assess the equivalence of the two testing kits [15], and the κ-value was interpreted as follows: ≥0.75, good; <0.75 and ≥0.4, moderate; <0.4, poor. We used the Z-test on a series of samples to analyze whether there were differences between the test kits. Differences with *p*-values of less than 0.05 were considered to be significant.

## Results

### Comparison of the results of all samples tested with the two kits

For clinical diagnostic TB cases, the sensitivity of the Trustline kit (61.3 %, 385/628) was significant higher than that of the Aupu kit (53.7 %, 337/628) (*p* < 0.05) (Table 3).

**Table 3:** Detection results from the 1,009 specimens using the two kits

Groups	Trustline kit, *n* (%)		Aupu kit, *n* (%)		Total
	Positive	Negative	Positive	Negative	
TB	385 (61.3)	243 (38.7)	337 (53.7)	291 (46.3)	628
Non-TB	53 (24.2)	166 (75.8)	31 (14.2)	188 (85.8)	219
Healthy controls	24 (14.8)	138 (85.2)	11 (6.8)	151 (93.2)	162
Total	462	547	379	630	1,009

### Comparison of the results between the bacteriological methods and the two kits

Among the 628 TB cases diagnosed clinically, of the 89 extrapulmonary TB patients, the sensitivities of the Trustline kit and the Aupu kit were 52.8 % (47/89) and 46.1 % (42/89), respectively, and of the 539 pulmonary TB patients, the sensitivities of smear staining, bacterial culture, the Trustline kit, and the Aupu kit were 41.0 %, 46.0 %, 62.7 %, and 54.9 %, respectively (Table 4). The sensitivities of the Trustline kit and the Aupu kit were significantly higher than that of the bacterial methods (*p* < 0.05). The sensitivity of the Trustline kit and the Aupu kit for testing sera antibody IgG was 62.2 %. The sensitivity of the Trustline kit was significantly higher than that of the Aupu kit (*p* < 0.05).

**Table 4:** Serum antibodies detection results from the 539 pulmonary tuberculosis (TB) cases using bacteriological methods and the two kits

Bacteriological methods	Trustline (IgG and IgM), *n* (%)		Trustline (IgG), *n* (%)		Aupu (IgG), *n* (%)		Total
	Positive	Negative	Positive	Negative	Positive	Negative	
Smear staining							
Positive	167 (75.6)	54 (24.4)	167 (75.6)	54 (24.4)	145 (65.6)	76 (34.4)	221
Negative	171 (53.8)	147 (46.2)	171 (53.8)	147 (46.2)	151 (47.5)	167 (52.5)	318
Bacterial culture							
Positive	190 (76.6)	58 (23.4)	190 (76.6)	58 (23.4)	165 (66.5)	83 (33.5)	248
Negative	148 (50.9)	143 (49.1)	148 (50.9)	143 (49.1)	131 (45.0)	160 (55.0)	291
Total of smear + culture							
Positive	239 (77.6)	69 (22.4)	239 (77.6)	69 (22.4)	209 (67.9)	99 (32.1)	308
Negative	99 (42.9)	132 (57.1)	96 (41.6)	135 (58.4)	87 (37.7)	144 (62.3)	231

The χ^2^ test was used to analyze the IgG antibody positive rate of the two kits compared with smear staining and bacterial culture, respectively, *p* < 0.05

### Comparison of the results from the two kits using bacteriological tests as the “gold standard”

Using the bacteriological test results as the “gold standard” to compare the results of the two kits, the sensitivity, specificity, PPV, NPV, and YI were 77.60 %, 79.8 %, 75.6 %, 81.9 %, and 0.574 by the Trustline kit, and 67.9 %, 88.9 %, 83.3 %, 77.4 %, and 0.568 by the Aupu kit, respectively (Table 5).

**Table 5:** Comparison of the detection results by the two kits using the bacteriological test results as the “gold standard” for TB diagnosis

Kits	Sensitivity	Specificity	PPV	NPV	YI
Trustline kit	77.6 % (239/308)	79.8 % (304/381)	75.6 % (239/316)	81.9 % (304/371)	0.574
Aupu kit	67.9 % (209/308)	88.9 % (339/381)	83.3 % (209/251)	77.4 % (339/438)	0.568
*p*-Value	<0.05	<0.05	>0.05	>0.05	

NPV, negative predictive value; PPV, positive predictive value; YI, Youden index

### Statistical analysis of the IgG antibody detection results from the two kits

Using the IgG results from the Aupu kit as a reference, the positive coincidence rate of the IgG results from the Trustline kit was 70.8 % (269/380), the negative coincidence rate was 70.4 % (443/629), and the total coincidence was 70.6 % (712/1,009) (Table 6). The equivalence test results gave a Cohen’s κ-value of 0.4, indicating moderate equivalence for IgG antibodies.

**Table 6:** Comparison of IgG results detected by the two kits

	Trustline kit		Total
Aupu kit	Positive	Negative	
Positive	269	111	380
Negative	186	443	629
Total	455	554	1,009

The χ^2^ test was used to analyze the IgG antibody-positive rate of the Trustline kit with respect to the Aupu kit: χ^2^ = 18.94, *p* = 0.000, *p* < 0.05

### Comparison of the IgG/IgM antibody detection results using the Trustline kit at the three hospitals

We compared the serum IgG/IgM antibody results from the Trustline kit from the three hospitals. The sensitivities were 61.8 %, 67.3 %, and 54.3 %; the specificities 61.1 %, 83.7 %, and 87.3 %; PPVs 81.0 %, 86.5 %, and 83.0 %; NPVs 37.4 %, 62.4 %, and 62.1 %; and YIs 0.23, 0.51, and 0.42, respectively, which were all within the acceptable ranges (Table 7).

**Table 7:** Comparison of the IgG/IgM antibody detection results using the Trustline kit at the three hospitals

Groups	Beijing Geriatric Hospital		Cangzhou Infectious Disease Hospital		General Hospital of Huabei Oil Field Company		Total	
	Positive	Negative	Positive	Negative	Positive	Negative	Positive	Negative
TB	149	92	134	65	102	86	385	243
Controls	35	55	21	108	21	141	77	304
Total	184	147	155	173	123	227	462	547

Control: non-TB + healthy; the χ^2^ test was used to analyze the IgG/IgM antibody-positive rate tested with the Trustline kit. For the case group, χ^2^ = 7.02, *p* = 0.030, *p* < 0.05, and for the control group, χ^2^ = 25.99, *p* = 0.000, *p* < 0.05

### Assessment of the IgM antibody detection results measured by the Trustline kit

The results showed that, of the 381 non-TB samples, 5 cases (1.3 %) were positive, while among the 628 TB samples, 30 cases (4.8 %) were positive. Of these 30 clinically diagnosed patients who were IgM-positive, two had acute hematogenous disseminated TB, 28 cases were secondary to TB, and 26 cases (87 %) were positive for both IgG and IgM (Table 8).

**Table 8:** IgM antibody detection results for the 1,009 cases tested using the Trustline kit

Case group	Trustline kit		Total
	Positive	Negative	
Case group	30	598	628
Control group	5	376	381
Total	35	974	1,009

The χ^2^ test was used to analyze the IgM antibody-positive rate between the case and control groups, χ^2^ = 8.50, *p* = 0.004, *p* < 0.05

## Discussion

Several reviews have provided performance evaluations of serological tests for rapid TB diagnosis [16–18]. A comprehensive review was published by Steingart et al. [17], who used a bivariate random effects meta-analysis to prespecify subgroups in order to address heterogeneity. They also summarized test performance by analyzing papers published from January 1, 1990 to June 29, 2010 after searching multiple databases. For anti-TB IgG, the pooled sensitivities were 76 % in smear-positive and 59 % in smear-negative patients, and the pooled specificities were 92 % and 91 %, respectively. Compared with ELISAs (pooled sensitivity, 60 %; pooled specificity, 98 %), immunochromatographic tests yielded lower pooled sensitivity (53 %) and comparable pooled specificity (98 %). In another study, Steingart et al. [18] used culture and clinical diagnosis methods as the reference standards. For pulmonary TB (eight test evaluations), commercial serological tests showed modest performance [diagnostic odds ratio (DOR) = 7.30], with a pooled sensitivity of 88 % and a pooled specificity of 50 %; for extrapulmonary TB (four test evaluations), the pooled sensitivity was less than 50 % and the pooled specificity was 93 %. In our current study using 1,009 specimens, according to the clinical diagnostic results, the pooled sensitivities and specificities of the Trustline kit and the Aupu kit were 61.3 %, 79.8 % and 53.7 %, 88.9 %, respectively (*p* < 0.05). After further analysis according to the results of smear staining and bacterial culture methods, the pooled sensitivities of the Trustline kit and the Aupu kit were 75.6 %, 65.6 % and 76.6 %, 66.5 %, repectively (Tables 4 and 5).

Analysis of the results of this clinical experiment gave a κ-value of 0.4 for IgG detection, indicating that there was no significant difference in the overall clinical diagnostic performance of the two kits. However, further analysis showed that, using the bacteriological test results as the “gold standard” for TB diagnosis to compare the results of the two kits, both the sensitivity and specificity differed between the two kits (both *p* < 0.05). The sensitivities of the Trustline kit and the Aupu kit for smear staining and bacterial culture negative were 53.8 %, 50.9 % and 47.5 %, 45.0 % respectively.

China has a high rate of TB infection (up to 44.5 % of the population) [1, 19], but only 5–10 % of those infected exhibit symptoms of active TB, and most of the remaining patients have latent infections [1, 20]. With the increased sensitivity of detection methods, the detection rate of latent infection will be increased, resulting in an increase in the false-positive rate and a decrease in specificity.

In the large number of clinical samples used in this study, the sensitivity of the Trustline kit was significantly better than that of the Aupu kit. In addition, the Trustline kit has the advantages of simple operation and short detection time, and can detect both anti-TB IgG and IgM antibodies simultaneously in human serum or plasma.

In summary, our data demonstrated that the Trustline TB IgG/IgM Rapid Test kit was more sensitive than the Aupu TB Ab (IgG) Colloidal Gold Test kit in the detection of TB antibodies in serum specimens. The Trustline TB IgG/IgM Rapid Test kit could detect both IgG and IgM in one test procedure and should be helpful for improving the detection rate of TB.
